# Adherence to antiretroviral treatment among children and adolescents in Tanzania: Comparison between pill count and viral load outcomes in a rural context of Mwanza region

**DOI:** 10.1371/journal.pone.0214014

**Published:** 2019-03-21

**Authors:** Giulia Martelli, Rosa Antonucci, Alphonsina Mukurasi, Henry Zepherine, Christiana Nöstlinger

**Affiliations:** 1 Department of Public Health, Institute of Tropical Medicine, Antwerp, Belgium; 2 Bukumbi Hospital, Mwanza, Tanzania; Centre de Recherche en Cancerologie de Lyon, FRANCE

## Abstract

**Background and objectives:**

Adherence to antiretroviral treatment is a key challenge for paediatric HIV care. Among children and adolescents living with HIV, lower levels of adherence have been reported compared to adults. Individual, caregiver-, health services-related and sociocultural factors were shown to impact on these outcomes. Study objectives were to assess adherence in a paediatric population in rural Tanzania comparing two measurement methods, and to investigate the association between virologic suppression and demographic, clinical, drug- and family-related factors.

**Methods:**

This cross-sectional study was conducted among children and adolescents enrolled in Bukumbi HIV Care and Treatment Clinic (Misungwi district, Mwanza region) in the north of Tanzania, where the HIV prevalence is 7.2%. Adherence was measured through viral load and pill count. Kappa statistics assessed the level of agreement between the methods; bivariate and multivariable analyses identified factors independently associated with virologic suppression.

**Results:**

N = 72 participants (n = 49 children; n = 23 adolescents) with a median age of eight years were enrolled. 62.5% and 65.3% of the individuals presented an optimal adherence according to viral load and pill count respectively, but among 40% viral load results diverged from the pill count method. In multivariable analysis, living outside Misungwi district and having CD4 counts above 500/μl were significantly associated with optimal adherence.

**Conclusion:**

Children and adolescents living with HIV in Mwanza show high rates of suboptimal adherence. The poor agreement between pill count and viral load results raises concerns about the interpretation of these measurements in clinical practice.

## Introduction

Children and adolescents accounted for approximately 10% of the 36.9 million people living with HIV worldwide in 2017, and about 80% came from Sub-Saharan Africa [1]. While the number of new HIV infections among children has declined by 35% between 2010 and 2017 [2], it remains unacceptably high despite significant progress in prevention of vertical HIV transmissions.

The introduction of effective antiretroviral (ART) treatment in the 1990’s drastically reduced AIDS-related mortality and morbidity. Adherence to ART became one of the most important determinants of long-term survival [3]. In addition, effective ART requiring optimal adherence also has become a major preventive strategy against the further spread of the disease on population level [4].

Adherence encounters special challenges in children and adolescents [3,5]; while children are dependent on caregivers for their health care, adolescents undergo a rapid physical and psychosocial phase of transition, resulting in development-specific issues and struggles [5,6]. In countries with low resources optimal levels of adherence have been found ranging from 49 to 100% among these populations [7]. This wide range is explained partially by different socio-cultural contexts, but also by the lack of standardized methods for measuring ART adherence. Drug adherence can be measured with subjective or objective methods [8]. Subjective methods include self- or caregiver-report and they tend to overestimate adherence, due to desirability or recall bias[9]. Objective measures consist of pharmacy refill pill count, electronic dose monitoring, drug detection in biologic samples and plasma HIV Viral load (VL); they are generally costly and sometimes less accepted by the patients [9]. Due to these advantages and disadvantages, a combination of multiple techniques has been suggested for better adherence assessment [10].

Adherence behaviour is a dynamic process influenced by multiple factors. They can be categorized as characteristics of the child/adolescent, the caregiver and the family, the health care providers, the society and culture and the medication [3,7,8]. These factors can interact with each other at different levels [11]: their association with optimal adherence is therefore complex. In addition, poverty, food insecurity[12], lack of comprehensive health services and limited access to health facilities [4] have been described as frequent challenges among young population living with HIV in middle and low-income countries [4,12–14].

Tanzania is one of the most HIV affected countries worldwide, with a prevalence of five percent [15]. The Joint United Nations Programme on HIV/AIDS (UNAIDS) estimated that at the end of 2017, 120.000 children were living with HIV in the country [16]. Some Tanzanian studies reported high rates of suboptimal adherence and/or virologic failure among children and adolescents [17–20]. Mwanza is a rural region in the North of Tanzania, where adherence among young populations living with HIV has not been broadly investigated.

The objective of this study was to assess the level of ART adherence among children and adolescents in one peripheral HIV centre of care in Mwanza region, by comparing two measurement methods, viral load determination and pill count. In addition, the demographical, clinical and social factors associated with optimal adherence were investigated.

## Materials and methods

### Study design

This cross-sectional study used quantitative routine monitoring data collected in an out-patient clinic for HIV, in Bukumbi CTC (Care and Treatment Clinic).

### Setting

Bukumbi CTC is located in Misungwi district, 40 km from the city of Mwanza. Mwanza region includes seven districts, predominantly rural [21]. Misungwi district hosts six CTCs [22]. According to the Population-based HIV Impact Assessment, Mwanza is one of the regions with the highest HIV prevalence in the country, reaching 7.2%; only 49.6% of the adult people living with HIV are virally suppressed [15].

At the end of 2016, N = 1116 individuals were followed-up at Bukumbi CTC; 1093 of them were on ART. In the clinic, n = 84 patients were < 18 years old. The clinic offers voluntary counselling and testing and medical consultations, provides first and second line ART for HIV-infected adults and children, as well as treatment of opportunistic infections and tuberculosis and baseline and follow-up laboratory investigations. Bukumbi CTC is partially financially supported by International Missionary Association, an Italian non-governmental organization.

### Participants

Study participants were all children and adolescents below 18 years and six months among the patients on ART, who were regularly followed-up at Bukumbi CTC for at least six months, and for whom of at least one VL determination was available for the period August 2016 to December 2017.

Patients were clinically managed according to Tanzanian National Guidelines [23]: according to the global “Test and Treat” strategy: from the end of 2016, ART was proposed to all patients irrespectively of their CD4 count or WHO stage. All patients, adults and children, were monitored through annual VL determination and biannual CD4 count.

### Data collection, variables and measurement

Demographical and clinical data were obtained through an electronic software (NACP/CTC2data 8^th^ version available for all Tanzanian CTCs). Information regarding health workers’ assessment through pill count were collected from patients’ files, based on the follow-up visits from May 2017 and August 2017.

Adherence was measured in two ways, choosing two proxy variables: VL measurements and pill counts. For VL measurements, results were categorized as < 1000 or ≥ 1000 copies/ml, reflecting optimal and suboptimal adherence respectively, according to the target indicated in the 90-90-90 UNAIDS goal and in the local guidelines [23]. Pill counts were performed by the health care workers, who assigned an adherence score based on pharmacy refill pill count and punctuality at each visit. Pill count rate derived from the equation: number of pills taken divided by total number of pills which were supposed to be taken over that period. The score was categorized as ≥ 95% or < 95% estimated ingested pills, reflecting optimal and suboptimal adherence respectively.

### Data analysis

Data were analysed using Epi Info 7. Descriptive analysis on demographic and clinical characteristics of the population was performed after data cleaning.

The agreement between the two adherence methods was assessed through Kappa statistics, considering Cohen’s coefficient as negligible if < 20%, weak from 21 to 40%, moderate from 41 to 60%, good from 61 to 80% and very good if > 80% [24].

The association between the outcome “viral suppression” and the different independent variables was analysed. The following independent variables were considered: child’s demographical characteristics (age, sex, district of residency), child’s clinical characteristics (CD4 count, WHO stage, previous history of tuberculosis, number of infections/year, nutritional status), child’s drug regimen characteristics (number tablets/day, type of ART regimen, number of previous ART regimen, duration of ART, preventive treatment with cotrimoxazole and with isoniazid), caregiver/family’s characteristics (type of relation caregiver-child, being double orphan, having or not HIV positive sibling/s, caregiver’s HIV serostatus, caregiver’s literacy, and caregiver’s ART adherence if HIV positive), and adherence measured by pill count. Nutritional status was assessed as follow: among subjects below five years moderate malnutrition was defined when weight-for-height z score was between– 1 and– 2 z score, severe malnutrition was defined below– 3 z-score; from 5 years and above, weight-for-height per age and sex (BMI-for-age and sex) was adopted. Therefore moderate malnutrition was defined between the third and the fifth percentile, while severe malnutrition was below the third percentile.

Performing the logistic regression, variables with a p-value ≤ 0.1 in the bivariate analysis were entered into the multivariable analysis. A backward stepwise regression analysis was applied, comparing the models through likelihood ratio test. The final model included all the variables with a p-value < 0.05, besides sex and age, considered as a priori possible confounders.

### Ethical concerns

The study was approved by the Institutional Review Board of the Institute of Tropical Medicine of Antwerp (on 30/03/2018). The principal researcher, a medical doctor previously employed in this setting, entirely took care of data extraction from the electronical database and from the patients’ files, respecting data confidentiality and patients’ anonymity. All data were managed anonymously, removing all personal identifiers, and analysed at aggregated level. All data were stored in password-protected files which only the researcher had access to. The Institutional Review Board of the Institute of Tropical Medicine of Antwerp waived the requirement for direct patient’s and parental consent because monitoring data were used.

## Results

### Characteristics of the studied population

**Fig 1:**
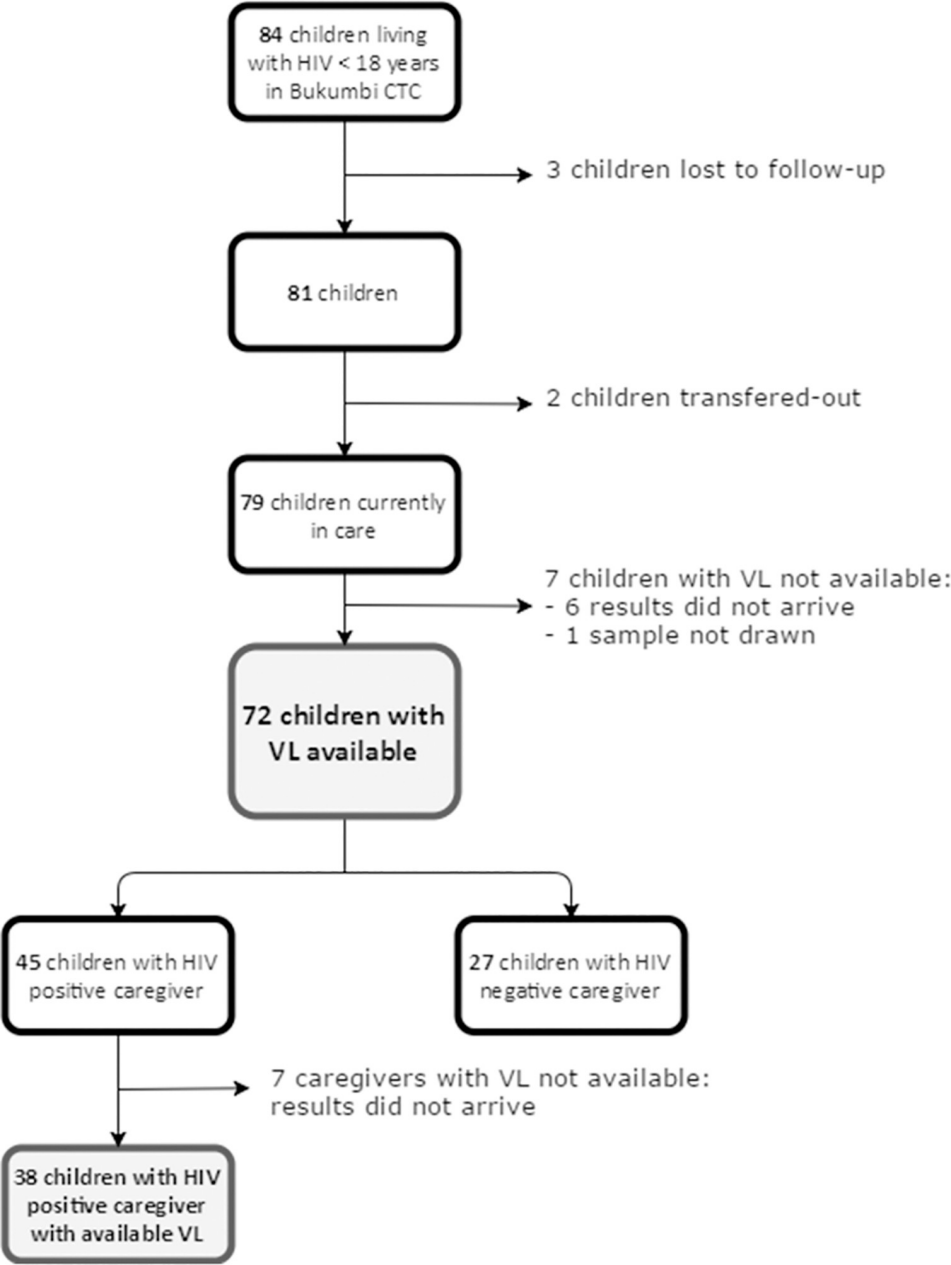
Flow diagram of enrolled population. CTC = Care and Treatment Clinic, VL = HIV viral load.

As shown in Fig 1, at the end of 2016, 84 individuals < 18 years and six month were enrolled in care at Bukumbi CTC; all of them were receiving ART. At the moment of data collection, three of them were lost to follow-up for longer than three months. Of the remaining 81, three were transferred-out to other districts. Seven children did not have VL results: six of them underwent blood sampling, but the results from the central laboratory did not come back, for one child it was not possible to draw a sufficient amount of blood. Finally, 72 children underwent a VL measurement from January 2017 to December 2017.

The characteristics of the study participants are described in Table 1. All individuals were vertically infected with HIV except for one, who was infected through a blood transfusion. Median age of participants was eight years (with n = 20 below five years); nearly half were male (female to male ratio = 1.18). Most of the subjects, i.e. 68.1% (49/72) were children (ten years and below) and 31.9% (23/72) were adolescents. The majority of the study respondents came from Misungwi district (77.8%, 56/72), the rest from neighbouring ones. The median time since diagnosis was four years and the median time receiving ART was three years. Most of the participants (62.5%, 45/62) had never changed their ART regimen. Most of the individuals were classified as WHO clinical stage III or II (respectively 52.8%, 38/72 and 30.5%, 22/72) and their median absolute CD4 count was 1285/μl (range 13 to 1578).

**Table 1:** Sociodemographic and clinical characteristics of the studied population.

Population Characteristics (N = 72)	% (n)
Gender:	
Male	45. 8 (33)
Female	54.2 (39)
Age:	
0–5	27.8 (20)
6–10	40.3 (29)
11–18	31.9 (23)
TB history:	
Negative	88.9 (64)
Positive	11.1 (8)
District of residence:	
Misungwi	77.8 (56)
Others	22.2 (16)
WHO stage:	
I	8.3 (6)
II	30.5 (22)
III	52.8 (38)
IV	8.4 (6)
ART current regimen:	
Non PI-based regimen:	76.4 (55)
3TC+AZT+NVP	47.2 (34)
3TC+AZT+EFV	8.3 (6)
ABC+3CT+EFV	20.8 (15)
PI-based regimens:	23.6 (17)
ABC+3TC+LPV/r	8.3 (6)
ABC+3TC+LPV/r	9.7 (7)
TDF+FDC+ATV/r	1.4 (1)
ABC+3TC+ATV/r	4.2 (3)
Time on ART:	
3–24 months	45.8 (33)
25 months -5 years	38.9 (28)
6 years-8 years	9.7 (7)
9 years-11 years	5.5 (4)
Number of ART tablet/day:	
≤2	41.7 (30)
2.5–4	13.9 (10)
4.5–6	38.9 (28)
≥6	5.6 (4)
Nutritional Status:	
Normal	62.5 (45)
Moderately malnourished	26.4 (19)
Severely malnourished	11.1 (8)
CD4 count:	
< 350/μl	9.7 (7)
≥ 350/μl	90.3 (65)
Relation with caregiver:	
Mother	51.4 (37)
Father	12.5 (9)
Other relatives	29.2 (21)
Others	6.9 (5)
HIV infected caregiver:	
No	37.5 (27)
Yes	62.5 (45)
Sibling/s affected with HIV:	
No	81.9 (59)
Yes	18.1 (13)
Caregiver’s literacy:	
No	40.3 (29)
Yes	59.7(43)

3TC = lamivudine, ABC = abacavir, ART = antiretroviral, ATV/r = atazanavir/ritonavir AZT = zidovudine, EFV = efavirenz, LPV/r = lopinavir/ritonavir, NVP = nevirapine, PI = protease inhibitors, TB = tuberculosis, TDF = tenofovir, WHO = World Health Organization

Roughly two thirds of the enrolled subjects, i.e. 62.5% (47/72) had at least one HIV positive caregiver and 18.1% (13/72) of them had HIV positive siblings. About a half of the caregivers were biological mothers (51.4%, 37/72); a third of the individuals, (36.1%, 26/72) were double orphans. More than half of the population, i.e. 59.7% (43/72) had at least one literate caregiver.

### Level of adherence and methods of measurement

Among the 72 analysed children and adolescents, 62.5% (45/72) achieved optimal adherence according to VL measurements, and 65.3% (47/72) according to pill count measurement.

**Fig 2:**
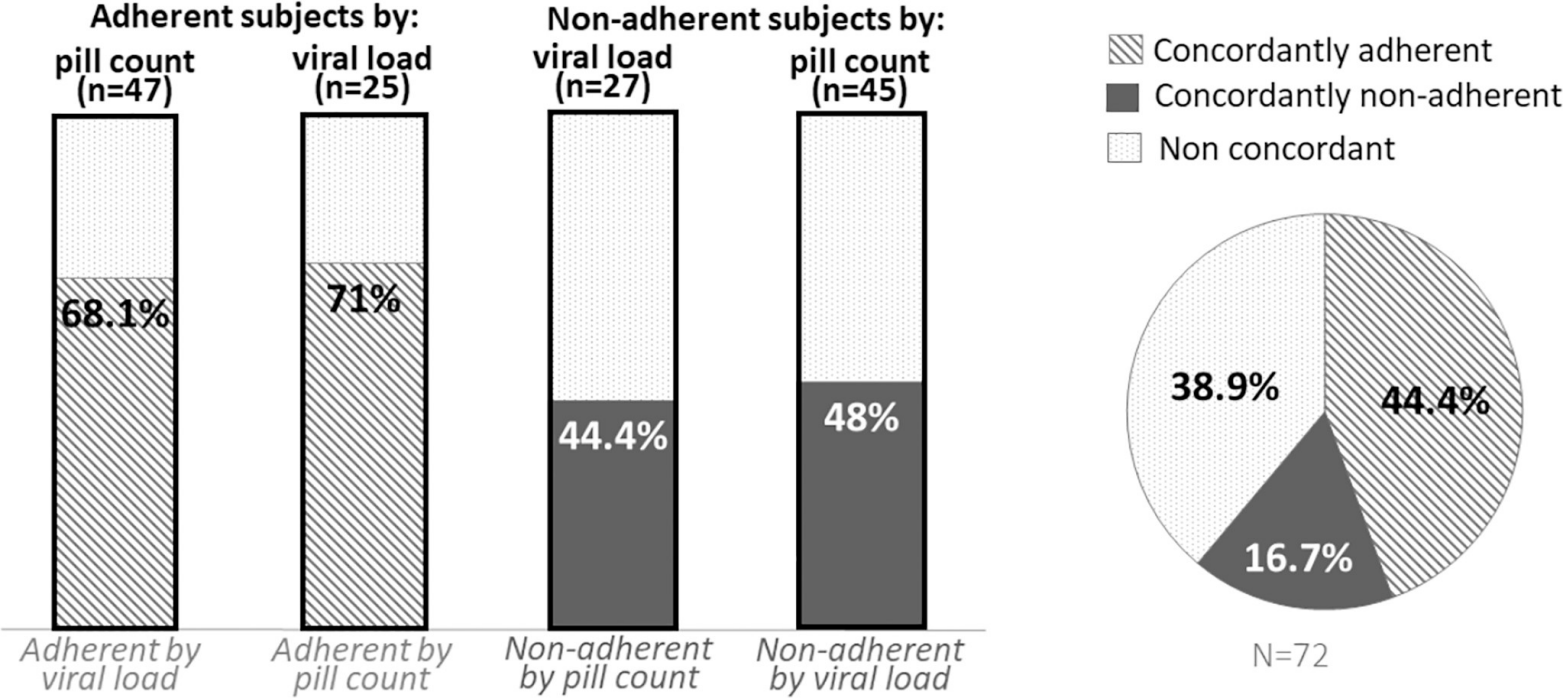
Agreement between the two measurement methods of adherence. The striped columns represent the percentage of agreement among adherent subjects, while the solid columns represent the percentage of agreement among the non-adherent ones. The pie chart on the right shows the percentages of agreement among the entire population.

The level of agreement between the two tests was very poor (Cohen’s Kappa coefficient = 16%, 95% C.I. -7.26–38.84). Fig 2 shows that only 68.1% (32/47) of the children classified with optimal adherence based on pill count presented suppressed viral load. This means that roughly one third of the children (31.9%, 15/47) who were considered by the health worker as adherent, presented detectable viral load (> 1000 copies/ml). Overall, as shown in Fig 2, 44.4% (32/72) of the individuals were defined as adherent by the two methods, while 16.7% (12/72) of the total cases were concordantly defined as non-adherent. Consequently, in 39.9% (28/72) of the cases VL and pill count assessments disagreed.

### Factors associated with viral suppression

Table 2 describes the results of bivariate and multivariable analyses, considering viral suppression (VL < 1000 copies/ml) as the primary outcome.

**Table 2:** Factors associated with viral suppression.

Factors	Bivariate analysis			Multivariable analysis (final model)		
	OR	95% confidence intervals	p-value	OR	95% confidence intervals	p-value
**Child’s demographic characteristics**						
Age *: < 5 years	**-**					
5–10 years	0.31	0.08–1.15	0.08	0.53	0.13–2.20	0.38
≥ 10 years	0.32	008–1.28	0.11	0.63	0.13–3.01	0.57
Sex *: F	-					
M	1.86	0.33–2.24	0.76	0.94	0.31–2.89	0.92
District **†**: Misungwi district	-					
Outside Misungwi district	5.64	1.17–27.17	0.03	17.24	1.64–181.29	0.02
**Child’s clinical characteristics**						
CD4 count **†**: < 500/μl	-					
≥ 500/μl	7.00	1.70–28.91	0.01	17.26	1.90–156.98	0.01
WHO stage: I or II	-					
III or IV	1.45	0.55–3.84	0.45			
History of tuberculosis	0.56	0.12–2.46	0.44			
Infections/year	0.78	0.78–1.06	0.12			
Nutritional status: Normal	-					
Moderately malnourished	1.44	0.46–4.50	0.53			
Severely malnourished	1.11	0.11–0.79	0.89			
**Child’s drug regimen characteristics**						
Number ART tablets/day	0.90	0.72–1.13	0.37			
ART regimen: Non PI-based	-					
PI-based	0.43	0.14–1.31	0.14			
Number previous ART regimens **§**	0.43	0.22–0.84	0.01			
Duration of ART (years) **§**	0.81	0.66–0.99	0.04			
Preventive treatment with cotrimoxazole	0.86	0.30–2.48	0.78			
Preventive treatment with isoniazid	1.47	0.35–6.26	0.60			
**Caregiver/family characteristics**						
HIV positive caregiver	1.60	0.60–4.26	0.35			
Having HIV positive siblings	0.64	0.19–2.17	0.48			
Relation caregiver-child: Mother	-					
Father	0.53	0.12-2-35	0.40			
Other relatives	0.47	0.15–1.41	0.18			
Others	0.63	0.09–4.34	0.64			
Mother or father	-					
Being double orphans	0.56	0.2–1.52	0.26			
Caregiver’s literacy: Illiterate caregiver	-					
Literate caregiver	1.03	0.39–2.73	0.95			
Caregiver’s ART adherence**: Suboptimal	-					
Optimal	0.98	0.16–6.00	0.98			
**Adherence (pill count)**						
≥ 95%	-					
< 95%	0.51	0.19–1.37	0.1823			

* Included in the final model of the multivariable analysis because a priori possible confounders

† Included in the final model of the multivariable analysis since p-value < 0.05

§ Significant in the bivariate analysis, but finally excluded since no more significant in the multivariable one

** Only among the ones who have HIV positive caregivers with available VL (N = 38)

ART = antiretroviral, VL = HIV viral load, OR = odd ratio, PI = protease inhibitors, WHO = World Health Organization

Based on bivariate analysis, among child’s demographical characteristics, living outside Misungwi district was the only variable significantly associated with viral suppression (OR = 5.64, 95% CI 1.17–27.17, p-value = 0.03). Children younger than five years and male subjects were more likely to have viral suppression compared to their counterparts, but this was statistically not significant.

Focusing on clinical features, having CD4 > 500/μl was significantly associated with viral suppression (OR = 7, 95% CI 1.70–28.91, p-value = 0.01). Having optimal adherence measured with VL was positively associated with having WHO stage of III or IV, but it was statistically not significant.

Among medication-related characteristics, longer time on ART and higher number of previous ART regimens were negatively associated with viral suppression (OR = 0.81, 95% CI 0.66–0.99, p-value = 0.04 and OR = 0.43, 95% CI 0.22–0.84, p-value = 0.01 respectively).

Among the factors related to the child’s family, having HIV positive siblings, having a caregiver other than the biological mother or being a double orphan were associated with viral replication, but these associations were statistically not significant. Children with HIV positive caregivers were more likely to be virally suppressed compared to those with HIV-negative caregivers, but again this was statistically not significant.

Finally, the individuals who took 95% or more of the medication according to the pill count, where more likely to have achieved viral suppression, but this association was statistically not significant.

In multivariable analysis, only living outside Misungwi district and having CD4 counts above 500/μl remained significantly associated with viral suppression (OR = 17.24, 95% CI 1.64–181.29, p-value = 0.02 and OR = 17.26, 95% CI 1.90–156.98, p-value = 0.02 respectively).

## Discussion

This is the first study addressing adherence in the specific context of Mwanza region, which has one of the highest HIV prevalence rates in Tanzania [15] and lacks data regarding adherence among young populations living with HIV. Comparing two adherence assessment methods provided an opportunity to overcome their respective limits and contributed to a better interpretation of the divergent results of the single procedures.

This study delineates low percentages of optimal adherence to antiretroviral treatment among children and adolescents living with HIV in the rural setting of Mwanza region: in Bukumbi CTC more than one third of the analysed population were not adherent to ART and the assessment routinely performed by health care workers based on pill count only partially reflected the results of viral load monitoring.

Two main factors were identified as being associated with suppressed vital load levels: children with a CD4 count above 500/μl and those coming from other districts than Misungwi were significantly more adherent compared to their counterparts.

While the available literature shows great variation in estimated levels of adherence among children and adolescents [7], the proportion of individuals virally suppressed found in Bukumbi CTC, i.e. 62.5%, is in line with the majority of Eastern African studies focusing on VL determination. Most of them found levels between 60 and 75% [17–19,25–27]. Only few authors reported a higher proportion of virally suppressed children, roughly 90%, but this value referred only to children more experienced with ART, while among the treatment-naive children rates of viral suppression were lower [28,29].

Most of the African studies which adopted pill count assessment found proportions of children and adolescents with optimal adherence to range between 35% [20] and 98.4% [30]. In Bukumbi CTC, 65.5% of the enrolled participants were adherent according to pill count calculation. Some studies reported reaching rates of more than 90%, but the analysed populations consisted of only children or preadolescents [25,26,30–34]. In parallel, other studies reported lower proportions of adherent individuals down to 35%, but they focused prevalently on adolescents only [20,35–38]. The worse adherence levels reported among adolescents compared to children [4,5] might explain these findings.

Although the results of the two methods adopted to measure adherence in Bukumbi might appear similar at first sight, the Cohen’s Kappa coefficient of 16% showed a very limited agreement. This reflects the lack of a standardized and accurate method to assess adherence. Additionally, the coefficient’s 95% confidence interval, although very broad (from -7.2 to 38.84) still fitted the definition of weak agreement (< 40% [24]). Despite the small sample size, we thus have sufficient evidence to conclude that the results of the two measurement methods widely diverge. Poor concordance between different adherence measurements was already observed in other East African studies [17,20,32–34,37,39,40]. Only few studies compared VL outcomes with pill count assessment [26,27,33,34]. Among them, two Ugandan studies found that virologic failure was more frequent among children with poor adherence measured with pill count [27,34]. On the contrary, Musiime et al. (2012) and Haberer et al. (2012) highlighted no significant association between pill count assessment and viral load, measured as binary and continuous variable respectively [26,33].

Children with CD4 counts higher than 500/μl showed significant better adherence, measured by viral load determination, than those with lower CD4 counts in the multivariable analysis. This is in line with other studies, which reported an association between CD4 count and virologic suppression [17–19,41]. Of course, this observation can be interpreted as a consequence, rather than a factor influencing adherence: high levels of adherence lead to better immunological and clinical outcomes. On the other hand, advanced WHO stages were described as facilitators for optimal adherence [37,42]. WHO staging is based only on the clinical findings at the time of the diagnosis of the disease and it does not change if the patient improves. Conversely, CD4 count gradually increases after ART initiation. Severe clinical conditions before starting the treatment and therefore a subsequently tangible improvement due to ART, might play a motivational role for a better adherence [43]. In our analysis individuals with WHO stage III or IV were more adherent, but that was not statistically significant.

Children living outside of the district of Bukumbi CTC were significantly more likely to be virally suppressed than their counterparts in the multivariable analysis. This is a surprising finding, since it contrasts with similar previous studies, which found shorter distances to the clinic to be associated with better adherence [44,45]. We measured only whether patients were living in- or outside Misungwi district, but not the distance house-clinic. A potential explanation for this finding may relate to HIV-related stigma: individuals coming from areas further away may intentionally avoid clinics nearby their home, showing some extra efforts in accessing treatment, which may influence their motivation. Also, other reasons not directly related to this variable might explain these findings: in Bukumbi CTC, for example, nutritional support was freely provided to malnourished patients, which could have attracted caregivers from other districts more concerned about their children’s health and therefore more adherent.

Concerning the relationship between age and adherence there is wide consensus in describing adolescence as the most challenging period for sustaining optimal adherence [4,5,14,46]. However, in our Tanzanian analysis, adolescents were found equally adherent to children younger than 10 years old. Also, no significant associations concerning the characteristics of the drug regimen were found. Other authors reported for example a positive association between better adherence and prophylactic treatment with cotrimoxazole [26,42,47] or with reduced pill burden-regimens [3].

Among the young population of Bukumbi CTC, being on ART for longer time and a higher number of previous ART regimens were found to be associated with virologic failure in the bivariate analysis, but this was not confirmed in the multivariable analysis. These findings are consistent with the results reported by Vreeman et al. (2008) [48]. However, in a recent large cohort of children from Tanzania, Uganda and Kenya, the same author described higher levels of adherence among those on ART for longer time [30]. Hence, whether being on ART for longer time is a facilitator or a barrier to optimal adherence remains unclear.

Having HIV positive caregivers has been described as a facilitator for optimal adherence [26]. In our study children with seropositive caregivers were more likely to be adherent, but this association was statistically not significant. Additionally, being single or double orphan or living with a caregiver different from biological parents reportedly are barriers to optimal adherence [30,35,38]; the study in Bukumbi highlighted higher risk of virologic failure among double orphans or those whose caregiver was not the mother, but these findings were statistically not significant. Finally, caregiver’s higher education was found to be associated with optimal adherence according to other African studies [44,49]; in our analysis having a literate or illiterate caregiver was not associated with optimal nor with suboptimal child’s adherence.

Several study limitations should be acknowledged. First of all, the small sample size of the enrolled paediatric population of Bukumbi CTC undermined the power of the analysis. Since our study was based on monitoring data, information regarding the factors associated with virologic suppression was limited to registered patients’ routine characteristics. The cross-sectional study design did not allow to draw any causal associations between investigated factors and adherence, nor to measure the evolution of adherence over time. An additional important limitation concerns the measurement of adherence. As described in literature, there is no gold standard to proper assess adherence to ART [31] and all the methods present some drawbacks: pill count assessment does not directly investigate the medicine ingested by the patient [8]; on the other hand, individuals with virologic failure might carry HIV drug-resistant strains and still be adherent [18].

In conclusion, this work provides a limited but accurate picture of an alarming scenario concerning virologic suppression among HIV positive children and adolescents in Mwanza region, Tanzania. From a public health perspective, it draws the attention to the need of setting up and promoting new preventive strategies and interventions addressed to children and adolescents and their caregivers, aiming at improving ART efficacy among these groups. Ultimately, this could contribute to reducing the transmission among the entire population.

An accurate assessment of adherence is a complex task to be chosen and evaluated carefully. The most widely adopted methods, such as self/caregiver reported or pill count, are often not consistent with the viral load determination measurement. The existing methods should be better validated, and the agreement between viral suppression and other methods, such drug concentration in biologic samples or electronic drug monitoring, considering patient autonomy while being conducive to reach fundamental goals of high ART adherence [50]. Such methods should be further investigated in order to better define an accurate and uniform approach to evaluate adherence among young populations in low resource settings.

Finally, factors associated with optimal or suboptimal adherence need to be investigated taking into account the context and the multiple levels at which they interact. Furthermore, a combination of quantitative and qualitative data, and their triangulation, could help to better explain the observed outcomes.

## Supporting information

S1 Dataset Dataset of the enrolled subjects. ID: identification number
TB: y = positive history of active tuberculosis, n = negative history of tuberculosis active
DiagnHIV: date of HIV diagnosis
District: 1 = Misungwi, 2 = others
ARVregimen:
    1 = lamivudine+zidovudine+nevirapine, 2 = lamivudine+zidovudine+efavirenz,
    3 = abacavir+lamivudine+efavirenz, 4 = abacavir+lamivudine+lopinavir/ritonavir (paediatric),
    5 = abacavir+lamivudine+lopinavir/ritonavir (adult),
    6 = tenofovir+emtricitabine+atazanavir/ritonavir,
    7 = abacavir+lamivudine+atazanavir/ritonavir
Nregimen: number of previous regimens
ARVStart: date which ARV was started
ARVtablet: number of ARV tablet/day
CTX: y = cotrimoxazole prophylaxis present, n = cotrimoxazole prophylaxis not present
Vitamine: y = vitamine supplementation present, n = vitamine supplementation not present
IPT: y = isoniazid preventive treatment present, n = isoniazid preventive treatment not present
Nutrition: 1 = normal, 2 = moderate malnourished, 3 = severe malnourished
Infection: number of infections that required antibiotics during the previous year
VLcat and VLRcat: 0 = < 20 copies/ml or not detectable VL, 1 = 20–1000 copies/ml VL,
    2 = ≥1000 copies/ml VL, 3 = not available
Adherence and AdherenceR: By pill count 1 = good (>95%), 2 = fair (85–95%), 3 = poor (<85%)
RelativeHIV: y = having at least one HIV caregiver, n = no HIV caregiver
Relation: With the caregiver 1 = Mother, 2 = Father, 3 = Relative, 4 = Other
Literacy: y = literate caregiver, n = illiterate caregiver
Siblings: y = having at least one sibling, n = no sibling
HIVSiblings: y = having at least one HIV sibling, n = no HIV sibling.
(XLS)
